# Public conformism with health regulation is crumbling as COVID-19 becomes a chronic threat: Repeated Cross-sectional Studies

**DOI:** 10.1186/s13584-023-00555-y

**Published:** 2023-02-03

**Authors:** Moran Bodas, Leora Wine, Kobi Peleg

**Affiliations:** 1 Department of Emergency and Disaster Management, School of Public Health, Sackler Faculty of Medicine, Tel-Aviv University, Ramat-Aviv, P.O.B. 39040, 6997801 Tel Aviv, Israel; 2 National Center for Trauma and Emergency Medicine Research, The Gertner Institute for Epidemiology and Health Policy Research, Sheba Medical Center, Tel-HaShomer, Israel

**Keywords:** COVID-19, Public trust, Threat perception, Conformity, Panic, Compliance

## Abstract

**Background:**

The purpose of this study is to analyze the long terms trends in public attitudes toward the COVID-19 pandemic and compliance with self-quarantine regulations.

**Methods:**

Repeated cross-sectional studies looking into data collected from nationally representative samples (N = 2568) of the adult population in Israel at five points in time representing the five morbidity waves of the COVID-19 pandemic. This study examined public trust in Israeli health regulations, levels of public panic, feelings of personal worry, and compliance with health regulations, specifically self-quarantine.

**Results:**

Public trust in health regulations in January 2022 is at an all-time low (25%) compared to the maximum value of nearly 75% measured in March 2020. While reported worry is steadily reducing, the perception of public panic is increasing. In earlier rounds, public compliance with self-quarantine was reported close to 100%; however, it has dropped to 38% by January 2022 when compensation is not assumed. Regression analysis suggests that trust is a major predictor of compliance with health regulations.

**Conclusions:**

The “fifth wave” of the COVID-19 pandemic brought about an all-time low in public trust in health regulations. The Israeli public, normally a highly compliant one, is showing signs of crumbling conformity.

## Background

On January 30, 2020, following the recommendations of the Emergency Committee, the World Health Organization (WHO) Director-General declared that the Coronavirus disease (COVID-19) constitutes a public health emergency of international concern [1]. Less than one month later, the first Israeli was diagnosed with COVID-19 [2]. The following month, in March 2020, the WHO officially declared COVID-19 to be a pandemic [3]. Nine months after the pandemic was declared, Israel administered its first vaccines to the public [4]. Two years later, as of 6 March 2022, there are more than 10,300 deaths and ~ 3.7 million confirmed cases in Israel constituting 38% of the total population [5]. As the fourth booster shot is readily available for adults over 60 and anyone 18 or older at high risk, more than 65% of Israelis have received all vaccination doses prescribed to them [6, 7]. At the peak of the fifth wave, on January 25, 2022, there were 101,095 new confirmed cases in Israel [6]. While Omicron has presented itself as a less severe variant of the COVID-19 pandemic, the virus does not show signs of disappearance and is a continued global threat to the public health, the social and economic well-being of humanity [8, 9].

Two years into the COVID-19 pandemic, experience and studies have shown that public behavior is a major contributor to disease spread increase or reduction. Behaviors such as wearing masks, maintaining lockdown or self-quarantine measures, personal hygiene, and social (physical) distancing are examples of health regulations that have been decreed. Public trust is an important factor in public compliance with these behaviors [10, 11]. At the beginning of this pandemic, adhering to these non-pharmaceutical interventions was especially critical as social behavior was the main tool to respond to the disease given that vaccinations were not introduced yet [12]. As the pandemic drags on, maintaining public trust to continue to comply with health regulations becomes more challenging over time as people have pandemic fatigue [13].

Many factors can influence one’s level of trust in government and health regulations, including target factors, such as news consumption, perceived threat, and level of COVID-19-related “fake news” [14], as well as some contextual factors, such as gender, age, and religious affiliation [15, 16]. In the United States, it was found that older, higher-income bearing people were more trusting of the government [15]. A cross-sectional study in Saudi Arabia found similar findings with women and older adults to be more trusting of government health regulations [17]. When looking specifically at precautionary measures relating to infectious diseases, women nurses are found to be more compliant than their male counterparts [18].

Israel is a country with a socialized medicine system. This means that the government runs the healthcare system. When it comes to trusting the healthcare system and trusting the government, it is not a given that citizens will trust or distrust them in the same manner. In Israel, studies show that people trust the healthcare system yet do not necessarily trust the government or the media [19]. This adds up to the political turmoil that Israel has been experiencing in recent years with multiple back-to-back elections and government instability [20]. While there was resentment and frustration that was directed towards the politicians, the government, and the media, this did not spill over to the healthcare system [19].

Another element found to be controlling public behavior during the COVID-19 pandemic was the perception of panic [21]. Group processes can affect individual behavior. When looking at panic on a communal level, people are more likely to be influenced by the beliefs of friends and family rather than the messages presented by government officials [22]. Studies show that the community groups that people belong to can also have a direct influence on how a threat is perceived [21, 23]. Increased threat perception by the group may lead to collective panic [21].

The purpose of this study is to analyze the trends in public attitudes toward the COVID-19 pandemic and compliance with self-quarantine regulations over the two years since the pandemic outbreak. As a working hypothesis, we propose that as the pandemic continues to drag on, people in Israel develop more pandemic fatigue and are less likely to comply with government health regulations, specifically concerning self-quarantine.

## Methods and materials

### Study design and setting

This study summarizes a series of repeated cross-sectional studies looking into data collected from nationally representative samples of the adult population in Israel at five points in time: late February 2020 as a baseline, Mid-March 2020 (peak of the 1st wave of COVID-19 outbreak in Israel), late August 2020 (during the 2nd wave), mid-January 2021 (peak of the 3rd wave), and mid-January 2022 (peak of the 5th wave). In addition, parts of the data included in this study were also collected during September 2021 (during the 4th wave). At each time point, a sample of > 500 participants was engaged to assess self-reported attitudes and compliance with health regulations during the COVID-19 pandemic. Variations in the circumstances at the different data collection points are noteworthy. In February 2020, as little as five cases of COVID-19 were confirmed, and over 5000 Israelis were under a self-quarantine at-home decree. By the peak of the first wave and the first national lockdown in March 2020, there were 677 confirmed COVID-19 cases, a few of those seriously ill, and nearly 60,000 Israelis in self-quarantine. At this point, 300 people died. The second wave of COVID-19 morbidity was seen in late August 2020, bringing about the second national lockdown in September 2020. During this time, a daily average of 21,000 active COVID-19 cases was observed. Roughly 60,000 Israelis were placed under a self-quarantine decree, and about 900 people died. Amidst the rise of the Beta variant causing the third wave and the third national lockdown in the middle of January 2021, the country counted ~ 600,000 confirmed COVID-19 cases, roughly ~ 70,000 per day, close to 1200 severe cases daily, and more than 4000 deaths. The country began a nationwide vaccination campaign shortly before the third wave in late December 2020. By the middle of January 2021, about 60% of risk groups (i.e. older than 60 and/or with comorbidity) were vaccinated. In late July 2021, the country was the first to offer a third dose (“booster”) to populations at risk. The fourth wave of COVID-19 morbidity (mostly due to the Delta variant of COVID-19) in August–September of 2021 saw the rise of a new government following several years of political deadlock. This new government resisted calls to enact a fourth national lockdown. At its peak, the fourth wave included nearly 10,000 daily active COVID-19 cases. Lastly, with the rise of the Omicron variant, Israel experienced its fifth wave of COVID-19 morbidity peaking at 76,000 confirmed cases daily and bringing about a so-called “silent” lockdown due to large portions of the population forced into self-quarantine (roughly 90,000 daily at the peak). In December 2021, the government began offering a fourth vaccine dose to populations at risk and in September 2022 a fifth dose that is adaptable to the Omicron variant. As of January 18th, 2023, 388,708 individuals were inoculated this fifth vaccines; Nearly 5000 active cases are reported daily [7].

### Population and sampling

The target population for this study was the adult population of the State of Israel. According to the OpenEpi ‘proportion’ sample size calculator [24], the minimum sample size for a population of nearly 10 million, with 95% confidence and a maximum marginal error of 5%, is 385 respondents. Nevertheless, the study was conducted using samples of at least 500 panelists in each round. Participants were recruited to this study through the *iPanel*, an online internet panel company that consists of over 100,000 members, representing all geographic and demographic sectors of the Israeli population. Sampling was done in a stratified method, based on the socio-demographic breakdown of the Israeli population as published by the Israeli Central Bureau of Statistics concerning age, gender, religiosity, and geographic zones. Informed consent was obtained from all participating panelists. The data was collected anonymously, following approval of the Ethics Committee of the Tel Aviv University (number 0003846-1 from September 2nd, 2021).

### Tools and variables

The study was based on a structured questionnaire that included items and indices that were developed specifically for this study and validated in previous rounds of data collection [13, 25]. The questionnaire included six items assessing public attitudes concerning the COVID-19 pandemic on a 5-point Likert scale, including news consumption (1 item), personal concern/worry (1 item), perception of public panic, and the media contribution to public panic (2 items), and attitudes toward public health regulations, namely, trust in health regulations (1 item) and whether taking criminal action against self-quarantine breaches will encourage more public compliance with regulations (1 item).

In addition, the questionnaire included two items assessing public compliance with health regulations on a nominal scale (Yes/No/Maybe/Do not know). The compliance measurement used the following wording: “Assuming you were requested by a medical officer to stay in self-quarantine and assuming the state will compensate you for lost wages, will you stay in self-quarantine?” Next, participants answered the same question phrased in the negative (“will not compensate”). In the March 2020 round, the questionnaire introduced another set of questions assessing whether the responder is currently in self-quarantine or ever was, on a nominal scale (Yes/No/Maybe/Do not know). Demographics were assessed by 10 items including gender, age, place of residence, family status, number of children, education, income, religion, religiosity, and immigration status.

### Statistical analysis

Descriptive statistics were used to analyze the characteristics of the sample. Chi-square tests were used to evaluate differences between samples. ANOVA test was used to assess differences in means across rounds. A logistic regression analysis was used to predict willingness to comply with health regulations without compensation for lost wages for the entire dataset, with data collection rounds serving as one of the independent factors introduced into the analysis. The dependent variable was re-categorized into two categories: Yes versus No (excluding responses of not sure and do not know). Variables were introduced into the regression analysis if they were associated with the dependent variable in the univariate analysis. The regression analysis was done both unadjusted and adjusted. All statistical analyses were performed using SPSS software version 27. *P* values lower than 0.05 were considered to be statistically significant.

## Results

The socio-demographic breakdown of the studied samples is provided in Table 1.

**Table 1:** Socio-demographic distribution (n (%)) of studied samples (Feb., Mar., Aug. 2020, Jan. 2021 and Jan. 2022)^+^

Variable	Feb. 2020 N = 563	Mar. 2020 N = 511	Aug. 2020 N = 511	Jan. 2021 N = 510	Jan. 2022 N = 503	*P* value
*Gender*						
Female	284 (50.4%)	262 (51.3%)	256 (50.1%)	264 (51.8%)	251 (49.9%)	0.972
Male	279 (49.6%)	249 (48.7%)	255 (49.9%)	246 (48.2%)	252 (50.1%)	
*Age*						
Average ± SD	39.57 ± 14.09	39.28 ± 14.24	39.37 ± 14.09	39.84 ± 14.30	39.81 ± 14.77	0.961
18–35	258 (45.8%)	239 (46.8%)	239 (46.8%)	231 (45.3%)	229 (45.5%)	0.972
36–55	213 (37.8%)	195 (38.2%)	185 (36.2%)	188 (36.9%)	180 (35.8%)	
56–70	86 (16.4%)	77 (15.1%)	87 (17.0%)	91 (17.8%)	94 (18.7%)	
*Religion*						
Jewish	456 (81.0%)	407 (79.6%)	407 (79.6%)	407 (79.8%)	403 (80.1%)	0.979
Other*	107 (19.0%)	104 (20.4%)	104 (20.4%)	103 (20.2%)	100 (19.9%)	
Religiosity**						
Secular	290 (63.6%)	267 (65.6%)	195 (47.9%)	188 (46.2%)	179 (35.6%)	< 0.001
Traditional	58 (12.7%)	52 (12.8%)	123 (30.2%)	121 (29.7%)	160 (31.8%)	
Religious	59 (12.9%)	51 (12.5%)	47 (11.5%)	48 (11.8%)	73 (14.5%)	
Ultra-Orthodox	49 (10.8%)	37 (9.1%)	42 (10.3%)	50 (9.8%)	91 (18.1%)	
*Place of residence*						
Haifa and North	199 (35.3%)	182 (35.6%)	176 (34.4%)	182 (35.7%)	124 (28.7%)	0.813
Tel-Aviv and Center	153 (27.2%)	143 (28.0%)	145 (28.4%)	133 (26.1%)	133 (30.8%)	
South and Coastline Plain	120 (21.3%)	102 (20.0%)	108 (21.1%)	100 (19.6%)	96 (22.2%)	
Greater Jerusalem	49 (8.7%)	46 (9.0%)	47 (9.2%)	48 (9.4%)	46 (9.1%)	
HaSharon Region	42 (7.5%)	38 (7.4%)	35 (6.8%)	47 (9.2%)	33 (6.6%)	
*Birth place*						
Israel	487 (86.4%)	464 (90.8%)	455 (89.0%)	460 (90.2%)	380 (88.0%)	0.171
Outside Israel	76 (13.6%)	47 (9.2%)	56 (11.0%)	50 (9.8%)	52 (12.0%)	
*Family status*						
Coupled	414 (73.5%)	359 (70.3%)	355 (69.5%)	340 (66.7%)	351 (69.8%)	0.189
Single	149 (26.5%)	152 (29.7%)	156 (30.5%)	170 (33.3%)	152 (30.2%)	
*Children*						
Yes	382 (67.9%)	304 (59.5%)	310 (60.7%)	305 (59.8%)	316 (62.8%)	0.026
No	181 (32.1%)	207 (40.5%)	201 (39.3%)	205(40.2%)	187 (37.2%)	
*Family size*						
Average ± SD	3.94 ± 1.78	3.89 ± 1.79	3.83 ± 1.70	3.94 ± 1.83	3.93 ± 2.00	0.854
1–2 members	137 (24.3%)	130 (25.7%)	127 (24.9%)	121 (23.7%)	143 (28.4%)	0.669
3–5 members	337 (59.9%)	297 (58.1%)	312 (61.1%)	312 (61.2%)	279 (55.5%)	
6 + members	89 (15.8%)	84 (16.4%)	72 (14.1%)	77 (15.1%)	81 (16.1%)	
*Education*						
< K-12	95 (16.9%)	84 (16.6%)	89 (17.4%)	91 (17.9%)	86 (17.1%)	0.007
K-12 diploma	110 (19.5%)	113 (22.1%)	105 (20.5%)	106 (20.8%)	105 (20.9%)	
Vocational	140 (24.9%)	111 (21.7%)	95 (18.6%)	110 (21.6%)	111 (22.1%)	
Bachelor's degree	166 (29.5%)	146 (28.6%)	167 (32.7%)	139 (27.3%)	139 (27.6%)	
Master's or above	52 (9.2%)	57 (11.2%)	55 (10.8%)	64 (12.5%)	62 (12.3%)	
*Income*						
Below average	230 (40.8%)	221 (43.3%)	233 (45.6%)	230 (45.1%)	217 (43.1%)	0.236
Average	117 (20.8%)	98 (19.3%)	108 (21.1%)	85 (16.7%)	91 (18.1%)	
Above average	162 (28.8%)	130 (25.4%)	118 (23.0%)	116 (22.8%)	141 (28.0%)	
Missing	54 (9.6%)	61 (12.0%)	52 (10.2%)	79 (15.5%)	54 (10.7%)	
*Employment*						
Part/fulltime employed	371 (65.9%)	332 (65.0%)	299 (58.5%)	294 (57.6%)	324 (64.4%)	< 0.001
Student	71 (12.6%)	71 (13.9%)	51 (10.0%)	50 (9.8%)	60 (11.9%)	
Unemployed	42 (7.5%)	37 (7.2%)	73 (14.3%)	86 (16.9%)	50 (9.9%)	
Self-employed	40 (7.1%)	34 (6.6%)	39 (7.6%)	27 (5.3%)	34 (6.8%)	
Retired	29 (5.2%)	33 (6.5%)	28 (5.5%)	22 (4.3%)	31 (6.2%)	
Missing	10 (1.8%)	4 (0.8%)	21 (4.1%)	31 (6.1%)	4 (0.8%)	
Self-quarantine***						
Were	–	44 (8.6%)	130 (25.4%)	220 (43.1%)	317 (63.0%)	< 0.001
Currently are	–	52 (10.2%)	22 (4.3%)	33 (6.5%)	45 (8.9%)	0.002

^+^Samples are mutually exclusive

*Includes: Muslims, Christians, and Druze

^**^Relevant to the Jewish portion of the samples only in all round except January 2022

^***^Data was collected from March 2020 onwards

The descriptive analysis of the data suggests several important findings. First, the data shows that public trust in health regulations in January 2022 is at an all-time low (Table 2). Public trust dropped from an average maximum of nearly 75% in March 2020 to less than 25% in January 2022. This drop is particularly registered among the ultra-orthodox Jews, with less than 6% in January 2022 following a peak of 78% in March 2020.

**Table 2:** Distribution [percentage of top two options from a 5-point Likert scale and means and standards deviations (SD)] of public attitudes toward COVID-19 related according to time in overall samples (N = 2568)

	% of top two options (“A lot” and “Very much”) and mean (± SD)					F* (*p* value)
	Feb. 2020 (N = 563)	Mar. 2020 (N = 511)	Aug. 2020 (N = 511)	Jan. 2021 (N = 510)	Jan. 2022 (N = 510)	
To what extent do you follow the news reports about the COVID-19 outbreak?	61.4% 3.76 ± 1.00	76.5% 4.13 ± 0.92	50.3% 3.48 ± 1.08	57.8% 3.61 ± 1.07	47.3% 3.41 ± 1.10	38.79 (< 0.001)
To what extent are you worried by the COVID-19 outbreak?	49.9% 3.55 ± 1.07	66.6% 3.91 ± 0.98	51.5% 3.49 ± 1.06	58.2% 3.61 ± 1.03	41.5% 3.29 ± 1.11	23.49 (< 0.001)
To what extent do you think the public is reacting in panic to the COVID-19 outbreak?	62.8% 3.78 ± 0.93	67.3% 3.95 ± 0.92	31.3% 3.16 ± 0.95	39.6% 3.25 ± 0.97	47.9% 3.46 ± 1.00	64.34 (< 0.001)
To what extent do you think the media is contributing to public concerns over COVID-19?	80.8% 4.14 ± 0.89	79.8% 4.17 ± 0.91	67.3% 3.84 ± 0.94	67.7% 3.87 ± 1.00	68.8% 3.92 ± 1.06	14.20 (< 0.001)
To what extent do you trust the public health instruction of the Ministry of Health during the COVID-19 outbreak?	52.8% 3.53 ± 1.05	74.5% 4.06 ± 0.87	38.0% 3.15 ± 1.04	43.0% 3.25 ± 1.06	24.9% 2.71 ± 1.15	118.19 (< 0.001)
To what extent do you think taking criminal action against individuals violating quarantine decree will increase compliance with MOH instructions?	69.2% 3.92 ± 1.04	78.7% 4.15 ± 0.91	65.5% 3.81 ± 1.07	62.7% 3.74 ± 1.09	46.3% 3.31 ± 1.21	42.12 (< 0.001)

*One-Way ANOVA

The data also shows significant trends in public perception of the panic in the overall population. Following a decrease from 67% in March 2020 to 31% in August 2020, the perception of public panic has been steadily rising to 40% in January 2021 and 48% in January 2022. Since August 2020, ~ 70% of responders have steadily associated public panic with media coverage of the COVID-19 pandemic.

In contrast, personal reported worry has fluctuated over time and is currently at an all-time low of 42% (Table 2). Similar trends are observed across the different sectors of the Israeli society, namely Arabs, Ultra-orthodox Jews, and other Jews. In January 2022, all sectors reported the lowest levels of worry, with 47% of Arabs reporting high worry, 45% of Jews (others than ultra-orthodox), and 23% of Ultra-orthodox Jews, compared with the peak values of 84%, 63% and 54% in March 2020 (respectively). See complete data in Table 2.

**Fig. 1:**
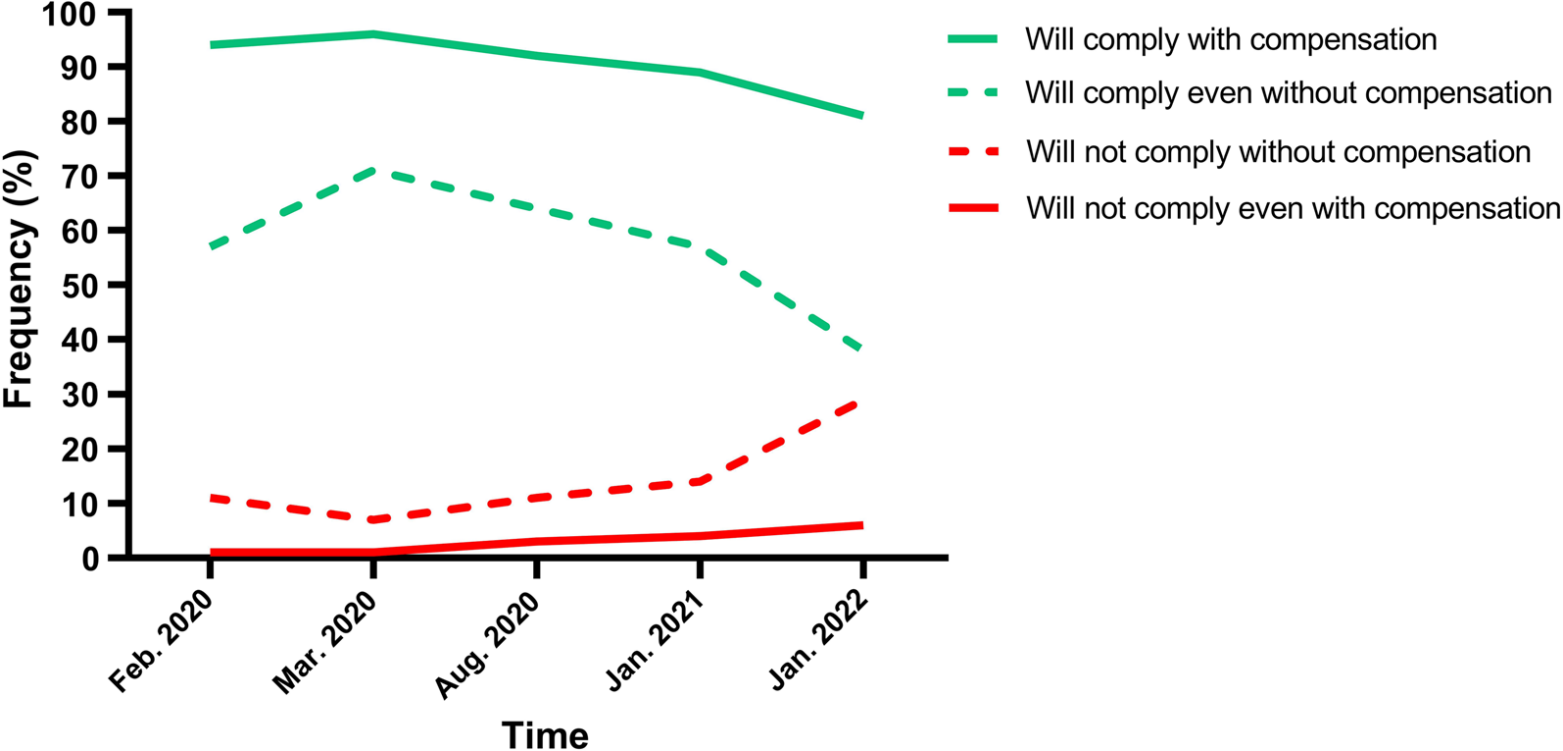
Trends in compliance with self-quarantine regulations according to whether compensation for lost wages is assumed or not

With regards to compliance with health regulations, specifically a self-quarantine decree, the data shows a major reduction in public willingness to comply. In the early stages of the COVID-19 pandemic (Feb–Mar 2020), compliance rates were nearing 100% when monetary compensation was assumed and 60–70% when not assumed. However, as the pandemic progresses, these numbers are changing. In January 2022, only 81% of responders said they would comply with health regulation assuming monetary compensation and as little as 38% when such compensation is not assumed. In parallel, the number of people bluntly reporting they will not comply with health regulation increased in January 2022 to 29% of responders who report they will not comply without monetary compensation and even 6% reporting intent of non-compliance even if compensation is offered. See complete data in Fig. 1.

To predict public compliance with health regulations, namely self-quarantine even without monetary compensation for lost wages, univariate and multivariate analyses were conducted. The univariate analysis suggests that women report higher compliance than men (62% compared to 53%), according to the Chi-square test (χ^2^ = 31.53, *df* = 2, *p* < 0.001). Older individuals aged 56–70 report higher compliance (69%) compared to those aged 36–55 (58%) and 18–35 (53%) (χ^2^ = 43.58, *df* = 4, *p* < 0.001). Individuals with a monthly income lower than average report more compliance (59%) compared with average earning (56%) and higher than average earning (57%) (χ^2^ = 12.08, *df* = 4, *p* = 0.017). Individuals with an academic degree (60%) more than non-academics (56%) (χ^2^ = 10.95, *df* = 2, *p* = 0.004). No statistical difference was observed on the basis of geographical location (*p* = 0.068), religiosity (*p* = 0.069), marital status (*p* = 0.966), parenting status (*p* = 0.072), and family size (*p* = 0.417).

Those willing to report offenders of self-quarantine also report higher compliance themselves (71%) compared with those who will not report offenders (χ^2^ = 340.74, *df* = 6, *p* < 0.001). Individuals who have yet to be in self-quarantine report higher compliance rates (62%) than those who already experienced self-quarantine (χ^2^ = 38.86, *df* = 2, *p* < 0.001). Compliant individuals also consume more news (3.83 ± 1.01) compared to non-compliant individuals (3.37 ± 1.15), according to independent Student *t*-test (t =  − 7.031, *df* = 512.94, *p* < 0.001). Compliant individuals are more worried compared to non-compliant individuals (3.78 ± 1.00 vs. 3.13 ± 1.17, respectively; t =  − 9.763, *df* = 502.01, *p* < 0.001) and express more trust in the health regulations (2.69 ± 1.17 vs. 3.58 ± 1.07, respectively; t =  − 13.264, *df* = 522.24, *p* < 0.001). Lastly, compliant individuals agree more with the assumption that punishment over breaches of self-quarantine will facilitate more public compliance (3.98 ± 1.00) compared to non-compliant individuals (3.29 ± 1.28) (t =  − 9.591, *df* = 479.28, *p* < 0.001).

However, the multivariate logistics regression shows that only a few of these factors retain their statistical significance as predictors of compliance with health regulation in the absence of monetary compensation. Primarily, compliance is predicted by increased trust in health regulations. See complete details in Table 3.

**Table 3:** Adjusted and unadjusted odds-ratio of intent to comply with self-isolation without compensation for lost wages according to correlated threat perception and socio-demographic variables (N = 2095)

Variable^a^	Unadjusted		Adjusted	
	OR	95% CI	OR	95% CI
*Round (Ref: Jan. 22)*				
Round 1 (Mar. 2020)	8.136	5.385, 12.294***	3.654	1.856, 7.193***
Round 2 (Aug. 2020)	4.335	3.039, 6.182***	2.729	1.619, 4.600***
Round 3 (Jan. 2021)	3.106	2.216, 4.355***	2.223	1.331, 3.711***
Age (cont.)	1.027	1.019, 1.036***	1.022	1.008, 1.037*
*Gender (Ref: Female)*				
Male	0.522	0.413, 0.660***	0.559	0.376, 0.831**
*Income (Ref: Lower than Avg.)*				
Average	0.645	0.456, 0.912*	0.594	0.345, 1.026
Higher than average	0.760	0.564, 1.026	0.845	0.524, 1.363
*Education (Ref: academics)*				
Non-academics	0.666	0.522, 0.848***	0.922	0.612, 1.389
*Been in self-quarantine (Ref: No)*				
Yes	0.452	0.350, 0.584***	0.888	0.584, 1.352
News consumption	1.491	1.340, 1.660***	1.119	0.911, 1.347
Worry over COVID-19	1.762	1.578, 1.967***	1.088	0.876, 1.352
Trust in MOH	2.012	1.805, 2.242***	1.326	1.089, 1.613**
Punishment efficacy	1.723	1.555, 1.910***	1.058	0.875, 1.278

*Ref* reference category, *OR* odds ratio, *CI* confidence interval, *MOH* Ministry of Health

^a^Only variables found to correlate with the dependent variable are shown. The categorization of the independent variables are as follows: Round (4 categories); Age (cont.); Gender (2 categories); Income (3 categories); Education (2 categories); Been in self-quarantine (2 categories); News consumption, Worry over COVID-19, Trust in MOH, and perception of efficacy of punishment of self-quarantine violations—ordinal from 1 (“not at all”) to 5 (“very much”)

*p value < 0.05 (two tailed), **p value < 0.01 (two tailed), ***p value < 0.001 (two tailed)

## Discussion

This study examined public trust in Israeli health regulations, levels of public panic, feelings of personal worry, and compliance with health regulations, specifically self-quarantine. This study found that the perception of public panic has steadily risen whereas personal worry has decreased. There are three main possible explanations for these findings. First, while the number of people who test as positive with the Omicron variant is higher than with the Delta variant of the fourth wave, the symptoms as a whole are less severe. Therefore, Omicron might be perceived as a lesser threat than previous variants by many experts and the government [26]. Second, as COVID-19 continues to have a dominant presence, people get used to the threat, normalize it in their lives, and become more resilient towards it [27]. Lastly, people may grow tired of the pandemic (a.k.a. “Pandemic Fatigue”) and the constantly changing health regulations [13]. These explanations are supported by the findings of this study which show a continued decline in worry over COVID-19, alongside the stable percentage (~ 70%) of responders who believe that the media is contributing to public panic.

As the COVID-19 pandemic continues, trust in the healthcare system and those who are delivering the messages becomes a more critical component in public compliance to follow these messages [13, 25]. Our findings show that trust in health regulations is at all times low. Regaining public trust in the health system, particularly at the decision-making level, is crucial for maintaining compliance with life-saving health regulations. When considering how effective a country can be in crisis management of the pandemic, one study conducted in Israel showed that the two variables that were directly related to the government’s effectiveness were citizens’ satisfaction with the public sector and their perception of participation in decision making [28]. Furthermore, it was suggested that during a crisis, people often think more about short-term satisfaction rather than long-term trust [28]. In both the short and long term, trusting a healthcare system and its messages remain important for public health and safety.

The data also shows a reduction in the public’s willingness to comply with health regulations, specifically self-quarantine. In earlier rounds, especially during the first national lockdown, public compliance was reported close to 100%; however, by January 2022 it has dropped to 81% when compensation for lost wages is assumed and goes down from 60 to 70% in earlier stages of the pandemic to 38% in January 2022 when compensation is not assumed. This decrease in willingness to comply shows that Israeli conformism, which was highly prevalent in previous COVID-19 morbidity waves [13, 25], is crumbling. People grow tired of the situation as pandemic fatigue becomes more prominent [13]. Specifically, regarding self-quarantine, there have been frequent changes to the instruction which can confuse [2]. When instructions are too difficult to follow, people may opt not to do them at all [29, 30]. This deviation from conformism could be the first of future disregard for public regulations. Such behavior can be risky and prove to be more challenging for future health regulations.

Accordingly, it is warranted to analyze the factors that are associated with non-compliance with health regulation, to determine risk groups. When breaking down compliance rates demographically, women in this study reported higher compliance than men, and older individuals, aged 56–70, report higher compliance compared to younger adults, aged 36–55. Similar findings have been reported in other studies where older adults and women have a higher tendency to comply with health regulations [2, 15]. One reason women may support the public sector more than men is that gender norms have a role to play in why women have a higher level of compliance [31–33]. Alternatively, women have become more dependent on the public sector for employment and therefore may view it more favorably [16].

There is more of a debate regarding the level of education and adherence to government regulations. Gozgor’s research showed that people with higher levels of education followed the government's instruction less [15]; however, Christensen and Laegreid [16] found the opposite to be true. Our data falls in line with Christensen and Laegreid’s study as it shows that those with an academic degree (60%) are slightly more likely to comply with government regulations than non-academics (56%). While the type of profession was not asked, typically jobs related to those requiring an academic degree may be more common to do remotely, whereas non-academic-based professions could be more difficult. As stated earlier, if people had to suffer financial loss without compensation, then they may be less likely to follow the rules. On the other hand, our data points out that individuals with monthly income lower than the average report more compliance. While these individuals also may rely on mobility to perform their jobs, the financial repercussions of non-compliance with government regulations could incur monetary fines that such individuals are not prepared to pay.

In terms of religion, public trust dropped the most in the ultra-orthodox sector, going from 78% in March 2020 to less than 6% in January 2022. The data may reflect political attitudes of distrust toward the government due to the formation of a new coalition in June 2021, in which the ultra-orthodox parties were excluded. Previous studies show clear associations between levels of trust in government organizations and compliance with health regulations [34, 35]. Moreover, it is known that religious leaders on both the community and national level are key figures regarding public trust and compliance with health regulations [36]. If these political and religious leaders are speaking against the government, this may be detrimental to the compliance of these audiences with health regulations issued by the government. In addition, in extreme religious groups, such as the Ultra-Orthodox Jews, there is a common distrust of science and by association also health authorities [36, 37], which is also non-beneficial in promoting public compliance.

In terms of policy, the findings of this study suggest numerous aspects to be considered by policy-makers. First, certain groups in the population are more inclined to lose trust in health regulations, namely young male adults and ultra-orthodox individuals. Understanding the unique needs and characteristics of these groups is warranted to tailor-make risk communication efforts and foster a sense of trust among these sections of the public. Second, the findings demonstrate the challenges associated with the transformation of COVID-19 from an acute pandemic to a chronic, ongoing epidemic. Specifically, the findings suggest a pandemic fatigue effect. Under these circumstances, policymakers are ought to seek new avenues for risk communication that fit a threat that is being normalized into day-to-day life. Though vastly different, the same mechanism of habituation exists with the terrorism threat in Israel, as people grow accustomed to the threat and develop their daily routine around it [38]. Simply raising awareness of the threat, when it is already normalized into daily life, will likely yield no fruit in promoting wanted public behavior (e.g., maintaining hygiene, getting vaccinated with booster jabs, etc.). Instead, a utilitarian approach for fostering certain behavior may be more appropriate, as proposed by other research exploring public compliance with preparedness for armed conflicts, another normalized threat in Israel [39]. Lastly, this study reiterates previous findings concerning the need to accommodate not just health-related concerns of the public, rather also financial ones [25, 40]. As the pandemic progresses and people's livelihoods are damaged, there seems to be less and less willingness by the public to comply with health regulations that may jeopardize households' income, such as self-quarantine. Policy makers must be attentive to all public needs, not just the health ones, if they are to provide an holistic solution to crisis management.

## Limitations

This study has several limitations. First, although efforts have been made to make the tool reliable through experts’ consultations, this study employs the use of non-validated tools that were designed for this research. Second, this study utilized an online panel to collect responses. While this option provided immediate access to a diverse sample of the population on a wide geographic distribution, it may limit the generalization of the conclusion to people with high digital literacy. Furthermore, self-reported compliance may not predict actual behavior. Third, as seen in Table 1, for some socio-demographic variables the samples were statistically different, namely religiosity and education. While the multivariate analysis is capable of overcoming these differences, conclusions should be drawn with caution. Fourth, although this study was able to capture a sizeable portion of unvaccinated participants, it is difficult to assess whether or not these participants are representative of this group. Lastly, this study was performed in Israel. Generalization of the conclusions to other populations should be done with caution.

## Conclusions

The findings of the current study, which looks over more than two years of the COVID-19 pandemic, highlight the importance of maintaining public trust for public compliance with health regulations. The “fifth wave” of the COVID-19 pandemic brought about an all-time low in public trust in health regulations. The Israeli public, normally a highly compliant one, is showing signs of crumbling conformity. Implications for future policy are discussed. The implications on future disaster management are plentiful. Decision-makers are urged to contemplate means to foster public trust, especially among risk groups, namely young male adults and ultra-orthodox Jews.

## Data Availability

The datasets used and/or analysed during the current study are available from the corresponding author on reasonable request.

## Abbreviations

ANOVA: Analysis of variance
COVID-19: Corona Virus Disease 2019
WHO: World Health Organization
